# Photothermal Agarose Microfabrication Technology for Collective Cell Migration Analysis

**DOI:** 10.3390/mi12091015

**Published:** 2021-08-26

**Authors:** Mitsuru Sentoku, Hiromichi Hashimoto, Kento Iida, Masaharu Endo, Kenji Yasuda

**Affiliations:** 1 Department of Pure and Applied Physics, Graduate School of Advanced Science and Engineering, Waseda University, Tokyo 169-8555, Japan; mitsen1019@fuji.waseda.jp (M.S.); ezailbroy_b7@fuji.waseda.jp (H.H.); jjj-lucky.roo706@ruri.waseda.jp (K.I.); 2 Department of Physics, School of Advanced Science and Engineering, Waseda University, Tokyo 169-8555, Japan; endo0223@akane.waseda.jp

**Keywords:** photothermal microfabrication, agarose, collective cell migration, epithelial cells

## Abstract

Agarose photothermal microfabrication technology is one of the micropatterning techniques that has the advantage of simple and flexible real-time fabrication even during the cultivation of cells. To examine the ability and limitation of the agarose microstructures, we investigated the collective epithelial cell migration behavior in two-dimensional agarose confined structures. Agarose microchannels from 10 to 211 micrometer width were fabricated with a spot heating of a focused 1480 nm wavelength infrared laser to the thin agarose layer coated on the cultivation dish after the cells occupied the reservoir. The collective cell migration velocity maintained constant regardless of their extension distance, whereas the width dependency of those velocities was maximized around 30 micrometer width and decreased both in the narrower and wider microchannels. The single-cell tracking revealed that the decrease of velocity in the narrower width was caused by the apparent increase of aspect ratio of cell shape (up to 8.9). In contrast, the decrease in the wider channels was mainly caused by the increase of the random walk-like behavior of component cells. The results confirmed the advantages of this method: (1) flexible fabrication without any pre-designing, (2) modification even during cultivation, and (3) the cells were confined in the agarose geometry.

## 1. Introduction

Recent advancements in microfabrication technologies enabled researchers to examine the dynamics of singe-cell-based cellular networks in the confined microstructures [1,2]. Microetching and microprinting are two major complementary approaches to form the confined spatial arrangement of cells. The former is based on the semiconductor etching technology with photolithography or three-dimensional polymer structures [3,4]. Their advantages are strict physical control of cell positions in the desired complex spaces of microstructures for assessing cell behaviors. However, the pre-designing of photomasks and complicated exposure–development procedures are required for this approach. Especially, the process of alignment adjustment of photomasks requires experienced techniques. In contrast, the latter microprinting technology is simple stamping of adhesion factors on the flat substrate to control the spatial distribution of cells [5,6]. Adding to the simplicity, stepwise activation of stamped thermoresponsive molecules for direct control of neurite elongation in pre-designed patterns during cultivation was recently reported [7]. However, in both technologies, the designs are pre-determined by the photomasks or stamps and thus renders difficult the preparation of changes in complex structures and additional micropatterning after the cells are seeded onto the microengineered environment.

One of the intriguing applications of these microfabrication technologies is for spatiotemporal regulation of collective cell migration for analysis. By using the microstructures or microprinted patterns, these structures can confine the cell migration in the artificially defined geometries. In recent years, various research groups have utilized microfabrication technologies to confine collective cell migration inside spatiotemporally restricted environments [8,9,10,11,12,13,14]. In their research, especially studies on two-dimensional sheets of epithelial cells have proposed at least two dominant mechanisms of the directional motion of epithelial cells [12]. One is that all the follower cells in the monolayer cells are regulated by the leading front cells [15,16,17,18]. Another factor is that the mechanical force transmission also contributes to maintaining the directional movement of cells [19]. For two-dimensional analysis of Madin–Darby canine kidney (MDCK) epithelial cell sheets, the width dependence of their propagation was analyzed in the stamped fibronectin patterns and concluded that the average migration velocity of the leading edge of the monolayer was linearly progressed and independent of distance in each width. Furthermore, the velocity varied depending on the width of the strips, increasing from 22 μm/h in the wider one (400 μm) to 39 μm/h in the narrower channel (20 μm) [9]. Similar results were reported featuring the migration of MDCK cells inside the cell-repellent polyethyleneglycol-dimethacrylate (PEG-DMA) microchannels ( 100 μm–300 μm) [11]. In their report, the single-cell tracking in the one-sided cell sheet revealed the two dominant factors of collective cell migration, a constant directed cell migration and a diffusion-like behavior, which is enhanced in a higher density gradient.

The technology was also applied for the fabrication of three-dimensional tubular structures for analysis of tubulogenesis and cell sheet extension. Xi et al. formed the tubular structures (25 μm to 250 μm in diameter) by solidifying polydimethylsiloxane (PDMS) around wires of varying diameters [14]. They also found that the average front velocity of the formed tubular MDCK cell sheets inside microtubes was constant and independent of the displacement distance. Furthermore, an increase in migration velocity was observed as the tube diameter increased, which was in contrast to the results of the investigation involving a two-dimensional sheet. As demonstrated in the above reports, the constructive approach can simplify the convoluted circumstances of the cell sheet environment and can facilitate the analysis for elucidating the undiscovered rules in collective cell migration behavior. However, all these achievements were limited to the cell migration behaviors in fixed confined structures. To understand the dynamic response of cell sheets after the sudden change of environment such as wound healing, technology with a flexible change of micropatterns during cultivation is needed.

To overcome the limitations of conventional microfabrication technologies, we have developed another microfabrication technology based on agarose photothermal etching. This technique can create micrometer-sized engineered structures by melting a portion of the agarose layer, regardless of whether the cells are present or not, without any photomasks nor complicated development–exposure procedures [20]. Exploiting the advantages of this method, we have been able to perform additional processing during cell culture, which was difficult using existing methods [21].

In this report, we applied the agarose microfabrication technology for the analysis of collective cell migration in two-dimensional confined structures and examined the ability and limitation of this technique. The advantages of this analysis method are that the starting condition of the extension of cells from the reservoir can be regulated by the agarose etching after the cells proliferated in the reservoir chamber, and also the wide range of size control for microfabrication of topographical constraints.

## 2. Materials and Methods

### 2.1. Cells

We experimented with mouse-derived endothelium-like cell line (MILE SVEN 1 (MS1); CRL-2279, ATCC, Manassas, VA, USA). The cells were cultured under 37 ${}^{{}^{\circ}}$C and 5% CO_2_ concentration and subcultured after about 5 days of incubation. The culture medium used throughout this experiment was Dulbecco’s modified Eagle’s medium (DMEM : Gibco, Thermo Fisher Scientific, Waltham, MA, USA) with 10% heat-inactivated fetal bovine serum (FBS: Gibco, Thermo Fisher Scientific, Waltham, MA, USA) and 100 U/mL penicillin-streptomycin (Gibco, Thermo Fisher Scientific, Waltham, MA, USA).

### 2.2. A 1480 nm Laser Photo-Thermal Agarose Microfabrication System

**Figure 1:**
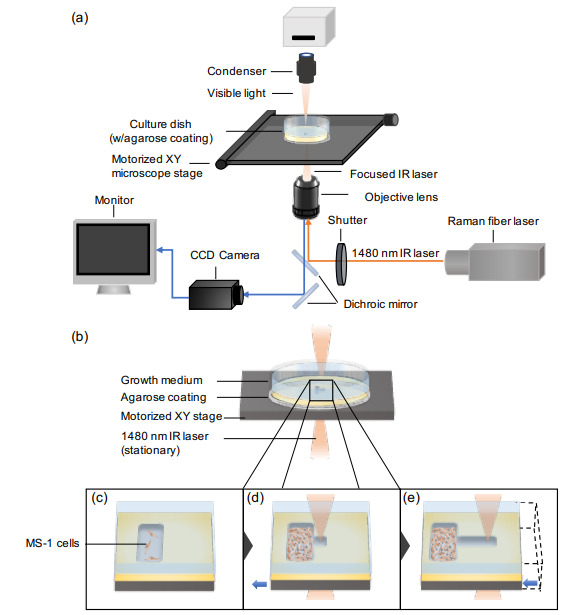
Photothermal agarose microfabrication technology for assessing collective cell dynamics. (**a**) Schematic of the agarose photo-thermal microfabrication device system. A 1480 nm wavelength infrared (IR) laser beam is focused by the objective lens onto the culture dish (w/agarose coating and water) and absorbed by the water present. The induced heat melts a portion of agarose coating locally, generating agarose removed areas where the cells can adhere. Controlling the motorized XY microscope stage manually or via program enables the fabrication of patterns accordingly to the user’s interest. (**b**) Magnified schematic of the agarose coated cultivation dish with laser irradiation for fabrication. (**c**–**e**) Method of photothermal microfabrication of straight microchannels on one-side of agarose. First, mouse-derived endothelium-like cell line, MILE SVEN 1 (MS-1) cells were seeded and adhered on the reservoir chamber (**c**). When the cells proliferated and became confluent in the reservoir, micorchannels were formed with focused IR laser (**d**). The shapes of microchannels were fabricated through the movement of XY microscope stage (**e**).

The agarose microfabrication system (schematic shown in Figure 1a) consists of a laser system (RLM-1-1480, IPG Laser, Oxford, MA, USA) equipped with an inverted bright-field microscope (IX70, Olympus, Tokyo, JAPAN). The 10X objective lens attached to the microscope focuses the 1480 nm wavelength laser beam emitted from the laser system. The laser beam irradiation was regulated by using a shutter controller (FSH-C, SIGMA KOKI, Tokyo, Japan) which can be controlled manually or by an automated regulation system modulated by a computer program. The motorized XY microscope stage is controlled by the joystick controller (FJ-401B, SIGMA KOKI, Tokyo, Japan) and operated through the feedback Stage Controller.

### 2.3. Microfabrication of Agarose Structures in the Thin-Coated Agarose Layer on the Cultivation Dish

A 35 mm tissue culture dish (AGC TECHNO GLASS, Shizuoka, Japan) was uniformly coated with 2.5 wt.% low melting point agarose (Promega, Madison, WI, USA) by using the spin coater (IH-D7, MIKASA, Tokyo, Japan) with the setting, 500 rpm for 3 s followed by 3000 rpm for 18 s. After coating a thin layer of agarose on the tissue culture dish and letting it rest for 5 minutes, the dish was filled with distilled water and placed on the microscope stage of the the photo-thermal agarose microfabrication system. Using the microfabrication system described above, the portion of agarose was dissolved by locally heating the water with the irradiation of the focused 1480 nm laser beam. The pattern of interest was microfabricated manually or by operating a Labview program that automatically controls the x and y direction of the motorized XY microscope stage and the shutter controller. The progress of the microfabrication was monitored through the Charged-Coupled Device (CCD) camera (CS-230, Olympus, Tokyo, Japan) which was projected to the connected monitor (PVM-14M1J, SONY, Tokyo, Japan). After the fabrication of agarose microstructures has been finished, the distilled water was washed and replaced with DMEM medium for cell culture. For the additional microfabrication during cultivation, the culture dish containing MS-1 cells and DMEM growth medium was set on the microscope stage of the system, and the alignment of the melting position of agarose microstructure was matched visually on the CCD camera image.

### 2.4. Cell Cultivation in Agarose Microstructures

A 1 mm (width) to 2 mm (length) dimension reservoir chamber was fabricated on the thin agarose layer coated on the 35 mm tissue culture dish using the agarose microfabrication system as described above. The dish was washed with DMEM medium three times and filled with 2 mL of fresh DMEM medium. Then, $3\times 10^{4}$ cells MS-1 cells were seeded on the tissue culture dish and incubated at 37 ${}^{{}^{\circ}}$C under 5% CO_2_. After about one hour of incubation, the cells over the reservoir chamber attached and adhered to the bottom surface of the cultivation dish. The other cells over the agarose coating region could not adhere and remained inside the supernatant. The DMEM medium was exchanged with a fresh DMEM medium to remove the excess cells in the supernatant from the dish. After the cells reached near confluence in the reservoir chamber, the microchannel constraints were additionally fabricated adjacent to the chamber in the agarose layer to guide cells from the reservoir chamber. Once the MS-1 cells migrated into the microchannels, time-lapse recording of the collective cell invasion was captured.

### 2.5. Time-Lapse Recording and Analysis of Cellular Dynamics

Time-lapse of every 10 min interval images of the cells inside the topographical constraints were taken by a CCD camera (CytoWatcher WSL-1800, ATTO, Tokyo, Japan) in the CO_2_ incubator (MCO-19AIC(UV), SANYO, Osaka, Japan) until they reach the end of the one-ended microchannels. From the obtained time-lapse images, the front cells, the observable leading edges, and particular single cells in the cell sheets were tracked by utilizing the MtrackJ plugin of Java language-based image processing program, ImageJ (NIH, Bethesda, MD, USA). By manually tracking the positions of the cells for image slices of 10 min intervals, the displacements and average migration velocities of the collective cellular flow inside two-dimensional confinement were measured. The aspect ratio of single cells in the cell sheets was also measured manually with ImageJ after creating binary images of the single cell. The value of the aspect ratio was evaluated as:

(1) $$Aspect\;ratio=\frac{Length\;of\;major\;axis}{Length\;of\;minor\;axis},$$

where the length of the major axis and the minor axis is the longest and shortest part of the binarized single-cell image.

The velocity auto-correlation and cross-correlation of cells in the channels are given by, $C\left(\Delta t\right)=\frac{1}{N}\sum_{i=1}^{N}\vec{v_{A}}\left(t_{i}\right)\;\cdot \;\vec{v_{B}}\left(t_{i}+\Delta t\right),$ where Δt is the given time difference, *N* is the total time steps, $v_{A}\vec{(}t_{i})$, and $v_{B}\vec{(}t_{i})$ are the velocity vector of cell A and cell B at time $t_{i}$, respectively. When cell A and cell B are same, C(Δt) is an auto-correlation. In contrast, when cell A and cell B are different, C(Δt) is a cross-correlation.

### 2.6. Statistical Analysis

All statistical values of propagation velocities are presented as mean ± standard deviation (S.D.) of 10 min interval recorded propagation velocities (unless stated otherwise). The normality of the acquired data was evaluated by the Shapiro–Wilk test. These propagation velocities were also evaluated using the F-test and following *t*-test. *p* < 0.05 was considered statistically significant. The Shapiro–Wilk test, F-test, and *t*-test were performed using R (R Foundation for Statistical Computing, Vienna, Austria).

## 3. Results

### 3.1. Epithelial Cell Cultivation in Agarose Microstructures

The schematic of the photo thermal agarose microfabrication system is presented in Figure 1a. The emission of the focused 1480 nm wavelength infrared (IR) laser is regulated by the shutter controller and irradiated via the objective lens. When the laser is exposed to the agarose-coated culture dish containing water (or DMEM medium), the water absorbs the laser and emits heat. Consequently, a portion of the agarose layer at the focused laser area (directly under the heat source) melts, enabling μm resolution microfabrication of patterns of interest. Finally, as the thin agarose layer coated on the cultivation dish prevents the adhesion of cells on the dish, the vascular endothelial cells can attach only to the exposed region of the tissue culture dish for proliferation.

The microfabrication process is illustrated schematically in Figure 1b–e, demonstrating the additional fabrication of a channel adjacent to the cell reservoir chamber. The reservoir chamber was prepared using the aforementioned device and MS-1 cells were seeded onto the culture dish (Figure 1c). The cells adhered only to the exposed reservoir region. After the cells proliferated and the chamber became nearly confluent, one-sided microchannels were fabricated on the lateral agarose walls by controlling the motorized XY stage (Figure 1d,e).

**Figure 2:**
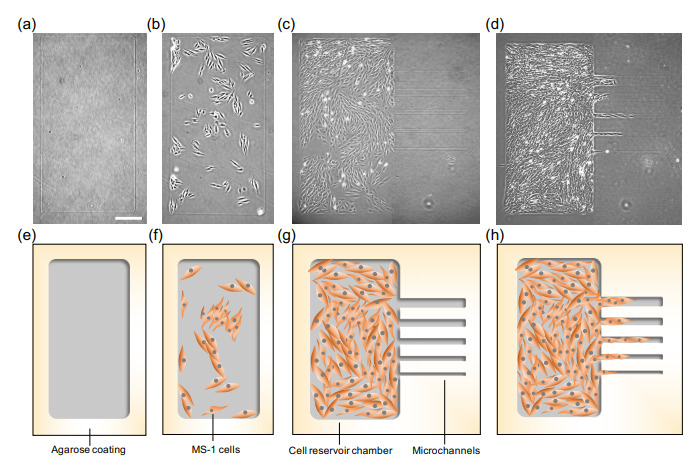
Application of agarose microfabrication technology for assessing collective cell dynamics in microchannels. (**a**–**d**) Micrographs of the stepwise microfabrication of channels for collective cell migration analysis. To display the whole image of the reservoir chamber and the microchannels as a micrograph, two micrographs (the left half of the chamber image and the right half of the chamber image) were taken and combined. (**e**–**h**) Schematics of Figures (**a**–**d**), respectively. (**a**,**e**) Agarose reservoir chamber was fabricated by melting a part of the agarose coating. (**b**,**f**) MS-1 cells were applied into the culture dish and adhered to the reservoir. (**c**,**g**) After MS-1 became nearly confluent, microchannels were formed in the agarose coating. (**d**,**h**) Cells were expanded into the microchannels. Bar: 300 μm.

The micrographs (Figure 2) represent the actual images of the microfabricated reservoir and microchannels. First, we fabricated the 1 × 2 mm reservoir chamber on the agarose layer of the dish (A micrograph, Figure 2a and its schematic drawing, Figure 2e). Then, $3\times 10^{4}$ cells MS-1 cells were seeded on the cultivation dish, and only the cells in the reservoir chamber adhered (Figure 2b,f). After the MS-1 cells proliferated and became confluent in the reservoir, microchannels were added to the reservoir chamber (Figure 2c,f). The culturing dish was placed on a timelapse capturing device inside the CO_2_ incubator to observe the cell migration phenomenon from the cell reservoir into the channels (Figure 2d,g).

**Figure 3:**
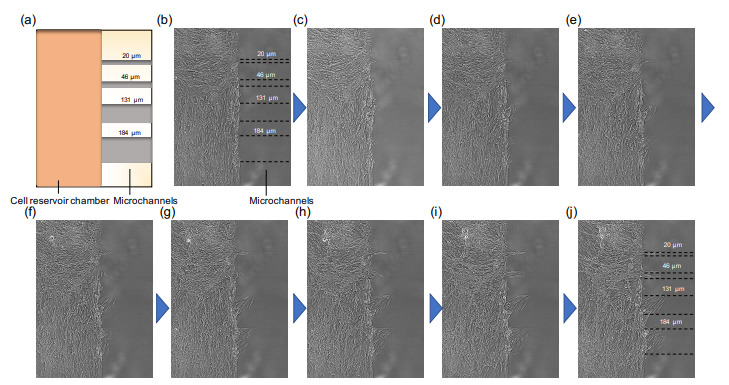
Time course images of collective migration of epithelial cells into the newly formed microchannels. (**a**) is the schematic drawing of the agarose micropatterns fabricated on the cultivation dish. Each 10 frame intervals of the supplement 10 min time-lapse movie is picked up from (**b**–**j**). (**b**) a micrograph just after microchannels fabricated using photothermal etching of focused IR laser; (**j**) 900 min later. Bars: 100 μm.

In this experiment, as demonstrated in Figure 2, we confirmed that we can form the desired collective cell migration patterns in the confined agarose structures. The MS-1 cells were captured within the walls of agarose etched patterns of the reservoir and microchannels, and no cells climbed over these walls. With this two-step microfabrication, we also can observe the expansion of MS-1 cells from the confluent cell reservoir to a series of newly added microchannels simultaneously (Figure 3 and Video S1). Moreover, during the additional microfabrication process of microchannels, the laser was emitted near the cells in the reservoir. However cell apoptosis was not observed during their movement into the microchannels (Figure 3 and Video S1). Thus, indicating that this photo-thermal etching technique is harmless to cells, allowing additional etching even after cells are seeded.

Adding to the ability and the advantage of the photothermal agarose microfabrication technique explained above, it should be noted that there is another major advantage about the simplicity and flexibility of this technology for collective cell migration analysis. All of these microfabrication procedures were operated manually while monitoring the process of photothermal etching of agarose layer with CCD camera images, which can acquire the 1480 nm wavelength light. The sizes, shapes, and positions of those microstructures are governed by the position of the microscope stage and the intensity of the IR laser, which are variables that are controllable to the user. In this experiment, we formed the microchannels from 10 μm to 211 μm in width with this simple spot-heating method. The photo-thermal etching technique introduced here is a direct fabrication method where the user can decide the dimension of the pattern during the fabrication process. Unlike the conventional microfabrication systems, it is unnecessary to prepare photomasks or microstamps in advance for this microfabrication. Therefore, this microfabrication technology requires no cleanroom circumstances and conventional microfabrication equipment.

### 3.2. Width Dependency of Collective Cell Migration

**Figure 4:**
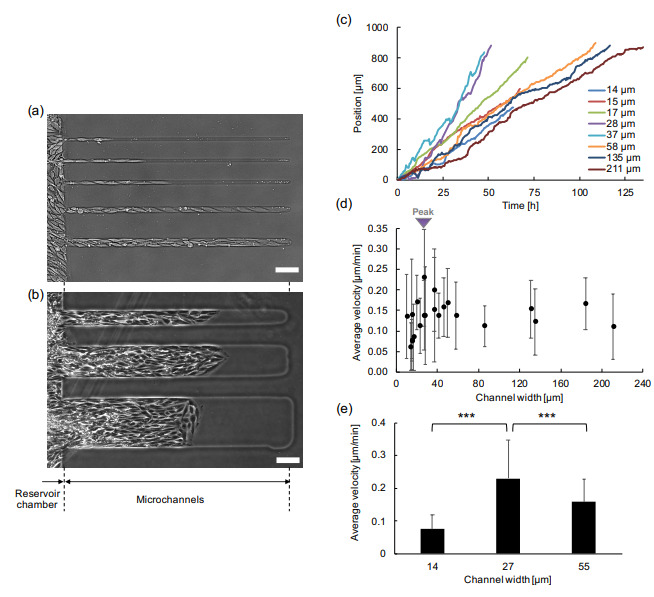
Collective cell migration in various width of agarose microchannels. (**a**) Micrograph of collective cell migration of MS-1 cells in 14.2, 15, 16.7, and 27.8 μm width agarose microchannels, respectively (from top to bottom). (**b**) Micrograph of collective cell migration of MS-1 cells in 58.0, 135, and 211 μm width agarose microchannels, respectively (from top to bottom). (**c**) Time course of collective cell migration in various widths of microchannels from 14 μm to 211 μm. The slopes represent the propagation velocity of their migrations. (**d**) Channel width dependence of migration velocity (mean ± S.D.). The peak velocity was observed around 30 μm width microchannels (inverse triangle). (**e**) Significance test of narrower width ( 14 μm), peak velocity width ( 27 μm), and wider width ( 55 μm) samples of (**d**). *** *p* < 0.001 unpaired Student’s *t*-test. Bars: 100 μm.

Collective cell migration was observed inside straight channel constraints of various widths ranging from 14 μm to 211 μm (Figure 4a,b). As exhibited in the micrographs, collective cell migration was observed in all the microchannels from 14.2 μm (top microchannel in Figure 4a) to 211 μm (bottom microchannel in Figure 4b) simultaneously. In the narrower microchannels, cells exhibited similar aligned elongated single cells in the direction of the channel, whereas the shapes of cells in the wider channels were varied.

First, we examined the propagation distance dependence of the migration velocity along the direction of each microchannel. The time course propagation distance from the reservoir for each channel is collectively expressed in Figure 4c. The slope of each sample was maintained constant including fluctuation regardless of the propagation distance from the reservoir at least in the range of 900 μm. Additionally, no tendency of deceleration was observed which indicates that the propagation velocities of collective cell migration in agarose microchannels were independent of the distance of the cell reservoir. Hence, this result suggests that the velocity is dominated mainly by the width of the microchannels rather than by the propagation distance. This phenomenon is similar to the previous report in the MDCK epithelial cell sheet migration on fibronectin stripes and PEG-DMA microchannel [9,12].

To reevaluate the influence of the spatial restriction on the cell migration phenomenon, the width dependence of the migration velocity was examined. The average migration velocities in various widths of microchannels are also expressed in Figure 4d. As shown in the graph, the fastest velocity was observed in microchannels around 30–40 μm in width becoming 0.230 μm/min, and the speed decreased both in narrower and wider microchannels. The decrease in the narrower microchannels demonstrates a linear correlation to the width of microchannels. In contrast, the decrease in the wider microchannels moderated and eventually saturated to a stable constant value.

Statistical significance of this one-peak and two-deceleration manner was also tested. As shown in Figure 4e, the collective cell migration velocities in 14 μm, 27 μm, and 55 μm width samples were tested as the narrower velocity, peak velocity, and wider velocity samples, respectively. The results showed that both the narrower velocity and the wider velocity from the peak velocity became slower, and they were statistically significant (*p* = $2.8\;\times \;10^{-5}$ , $2.2\;\times \;10^{-16}$ , respectively).

To explain this one-peak and two-deceleration manner, at least two fluid-like factors were hypothesized to contribute to this velocity reduction, such as (1) cell morphology in compressed channels in narrower microchannels, and (2) decrease due to the increase of random walk-like behavior in wider microchannels. To further investigate the possibility of the above two factors, single-cell tracking was conducted for cells inside narrower microchannels and wider microchannels.

### 3.3. Single Cell Tracking in the Collective Cell Migration in Narrower Microchannels

**Figure 5:**
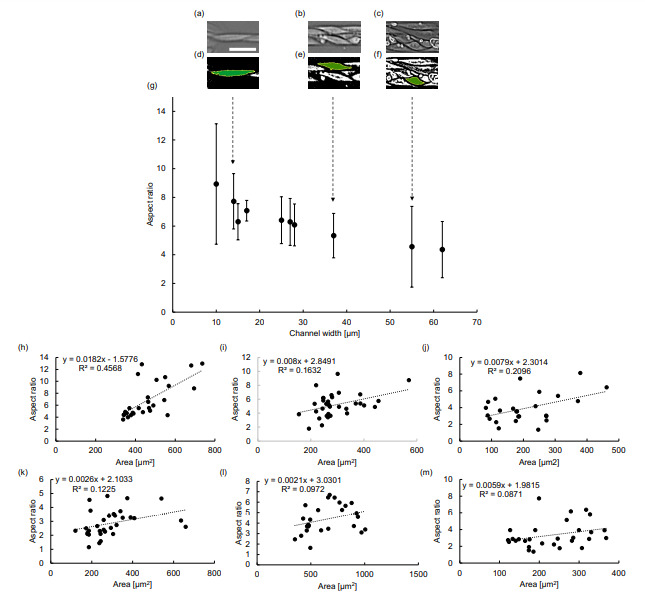
Single cell morphology characteristics in narrower microchannels. (**a**–**c**) Examples of acquired images of single cells in microchannels. Bar: 50 μm. (**d**–**f**) Binarized images of single cells of (**a**–**c**), respectively. (**g**) Microchannel width-dependence of aspect ratio of cell shapes (mean ± S.D.). In comparison to the wider channels, the cells in the narrower channels maintained elongated cell shapes. (**h**–**j**) Relationship between area and aspect ratio of the cells inside the 14 μm , 37 μm, and 55 μm channels, respectively. Additionally, area and aspect ratio dependence is also analyzed for 211 μm channel, where (**k**) is the inner follower cells and (**l**) is the cells in front half of the channel. (**m**) The area and aspect ratio analysis of cells inside the cell reservoir area.

As exemplified in Figure 5a, the shape of single cells in the microchannels in the narrower channels was significantly different from the cells in the wider channels and the reservoir. Hence, we examined the relationship between cell morphology and microchannel width, particularly in the narrower microchannels.

Analysis of single cells inside the narrower microchannels was conducted to identify the factor of morphology causing the migration velocity reduction in channel widths smaller than 30 μm. As demonstrated in Figure 5a–c, single-cell images were acquired from the micrographs of migrating cells in microchannels. Then, those cell images were binarized to estimate the aspect ratio of those cells (Figure 5d–f). As demonstrated in Figure 5g, the cell shape analysis revealed an approximately inverse linear correlation between the channel width and aspect ratio of the cells. In our results, the mean values of aspect ratio 4.3 in 60 μm width increased gradually and finally reached 8.9 in 10 μm width. The aspect ratio was greater for cells propagating inside channels of narrower width, indicating that cells were more elongated as the channel width decreased. As the peak velocity was achieved in channels around 30 μm, it is possible that an optimal aspect ratio exists in which the cell cohort can migrate the fastest. Finally, the excessive elongation of cell shapes appears to be an origin of the deceleration of propagation velocity.

The area analysis was also conducted to investigate the relationship between the area and the aspect ratio of cells inside different channels (Figure 5h–m). In comparison to the wider areas, such as the 211 μm channel (Figure 5k–l) and cells inside cell reservoir chamber (Figure 5m), the narrower channels of 14 μm (Figure 5h), 37 μm (Figure 5i), and 55 μm (Figure 5j) demonstrate stronger correlation between the aspect ratio and the size of cells in narrower micorochannels.

It is known that cell cohorts exhibit contraction–elongation-like migration mechanisms inside narrower channels, inducing a faster migration speed [9]. It is consistent with our results in microchannels wider than 30 μm. However, in excessively narrow channels below the width peak such as 30 μm, our results suggest that cells’ aspect ratio increased significantly, resulting in the decrease in velocity.

**Figure 6:**
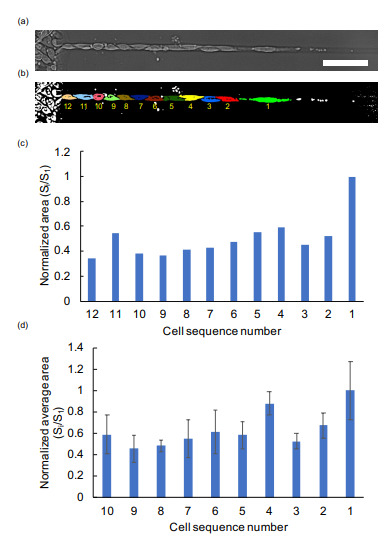
Distribution of aligned single cells in a 10 μm microchannel. (**a**) Micrograph image of collective cell migration in 10 μm microchannel. Bar: 100 μm. (**b**) Binarized image of single cells of (**a**). Each color and the number represents a single cell. (**c**) Normalized area distribution of cells of (**a**). (**d**) Normalized average area distribution of cells in 10 μm microchannels (*n* = 3). (Bars: mean length of cells; error bars: S.D. of length).

Figure 6 exemplifies one of the size distributions of the one-dimensionally organized cells within the 10 μm width microchannel. The length of the front cell was extraordinarily long, having the maximum aspect ratio. However, the length of the follower cells seems to form the smaller cells. In the previous two-dimensional cell sheet studies, it was reported that the advancing edge of the front cells protrude as leader cells and mechanically attached follower cells migrated with a density gradient [15,16,17,18]. Here, the results indicate even the linearly organized single cells, which maintain contact solely at the rear and front edges, follow the protruding leader cell and form shorter cells behind.

### 3.4. Single Cell Tracking in the Collective Cell Migration in Wider Microchannels

**Figure 7:**
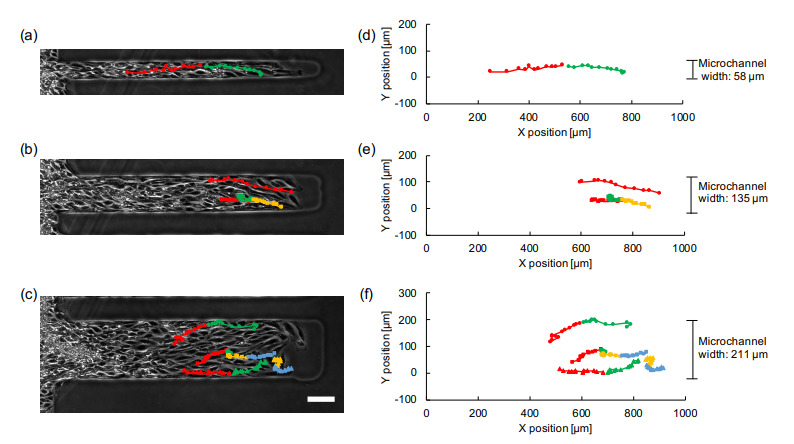
Tracking of single cells inside collective migration in wider microchannels. (**a**–**c**) Micrographs of collective cell migration with superimposed single cell tracking in wider microchannels having 58 μm (**a**), 135 μm (**b**), and 211 μm (**c**) widths. Each plot shows the position of the cell tracing in 1 h interval and lines connecting those plots to indicate the tracking of single cells in the microchambers. The colors of plots and lines of each tracking was changed every 12 plots from red, green, yellow, to blue, respectively, to emphasize the temporal aspect of these tracking intervals. (**d**–**f**) Graphs of those plots and tracking lines of (**a**–**c**), respectively. X-direction represents the direction of microchannel length, and Y-direction represents the direction perpendicular to the direction of microchannel length or the direction of microchannel width. Bar: 100 μm.

In the wider channels, the tracking of a single cell in both the vertical and horizontal directions was conducted. Figure 7 shows the tracking of single cells in 58 μm, 135 μm, and 211 μm width microchannels. Each plot represents the position of the tracking single cell in an hour interval, and the color of the plots and the connecting lines were altered every 12 plots. As demonstrated in Figure 7, the cells propagating inside wider microchannels exhibited more fluctuation both in time and trajectory in contrast to the narrower channels such as 58 μm, in which the cells proceeded straight forward with longer strokes between neighboring two plots.

**Figure 8:**
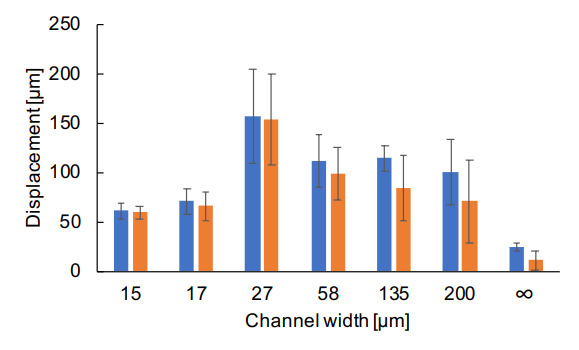
Evaluation of single cells’ total displacement distance and end-to-end distance in various microchannels. The tracking of single cells inside collective migration was computed to acquire the root-mean-square total displacement distance (blue bars) and the root-mean-square end-to-end distance (orange bars). In channel width axis, *∞* represents the tracking of isolated single cells in the free space without spatial constraints on the cultivation dish.

To clarify the contribution of fluctuation in collective cell migration velocity, the root-mean-square end-to-end distance and the root-mean-square total displacement distance of single cells in each microchannel were calculated and compared. Figure 8 represents the results of the microchannel width dependence. As demonstrated in the graph, the end-to-end distance and the total displacement distance in the narrower microchannels were almost equal. This indicates that the cells migrated straight forward with only minor fluctuation in displacement. In contrast, the values of these two indices deviated in the wider channels, suggesting the contribution of randomness in the movement direction during the propagation of cells. As a result, the random walk-like behavior in the wider channels causes the migration velocity to decrease overall.

Note that the total displacement distance itself has a peak at 27 μm width in this graph. As aforementioned, the decrease in both the end-to-end and total displacement distance is observed in the narrower microchannels. However, in the wider channels, the total displacement distance converged to a constant value after decreasing from the peak value, whereas the end-to-end distance decreased continuously according to the increase of the width of microchannels. The tendency can be explained by two reasons. The constant values of the total displacement distance indicate the step size of displacement in single cells is constant. Under this constant condition, the decrease in the end-to-end distance occurs when the free space of displacement increases. Applying this interpretation, the results of isolated single cells in free space can be interpreted that the step size of displacement of isolated single cells was smaller than that of the collective cell migration and the direction of displacement of isolated single cells was completely random in space.

**Figure 9:**
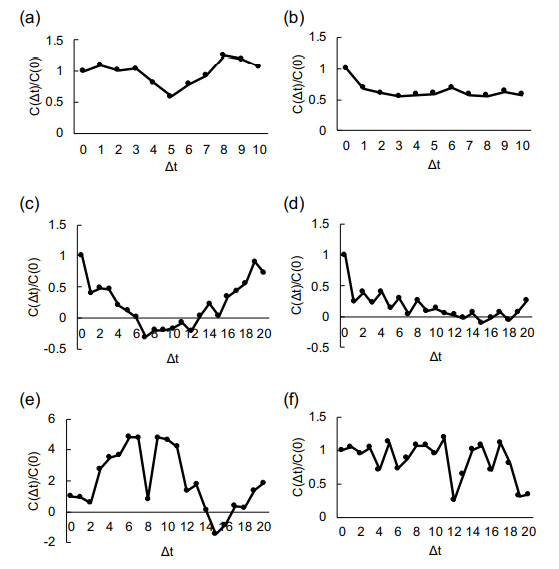
The velocity auto-correlation of cells in the microchannels. (**a**–**d**) Auto-correlation of the velocities of cells traced in 10 μm (see Figure 6), 58 μm (Figure 7a), 135 μm (Figure 7b), and 211 μm width channels (Figure 7c), respectively. (**e**,**f**) Cross-correlation of two cells in each of 135 μm (Figure 7b), and 211 μm (Figure 7b) channels, respectively. The auto- and cross-correlation values C(dt) were normalized by C(0).

Figure 9 shows the velocity auto-correlation of cells in the various widths of microchannels. As described in auto-correlation graphs, the cells contain the tendency of coordinated collective migration behavior rather than the simple random walk-like diffusive movement. Cross-correlation results also indicate the coordinated behavior of two-component cells in the wider microchannels.

### 3.5. The Strengths and Limitations of Photothermal Agarose Microfabrication Technology

As described above, agarose microfabrication technology was applied for collective cell migration study. The advantages and limitations of this technology are summarized in Table 1. As the minimum size of the focus area of the infrared laser is limited by the diffraction limit of optics, the spatial resolution of photothermal agarose microfabrication is determined by the wavelength of light.

Recently, we improved the photothermal agarose microfabrication technology by exploiting the conversion of IR laser into absorption heat on a 0.7 µm diameter platinum-coated microneedle for more precise microfabrication. This method can overcome and complement the potential limitations of photothermal microfabrication, and the diffraction limit of optics [21].

A quick and simple microfabrication procedure is also another advantage of this technology. Fabrication velocity is in the range of 100 μm/s for removing the agarose layer. Hence, when we set the spot size at 10 μm, we can form agarose microstructures in $10^{3}$ μm^2^/s resolution.

As this low melting point agarose gel changed into sol state at 60 ${}^{{}^{\circ}}$C, the focused IR beam does not damage the surface of the cultivation dish under such a weak intensity. In our previous studies, the collagen or poly-D-lysine layer on the dish was not damaged by the photothermal fabrication procedure and worked effectively for cardiomyocyte network study or neuronal network formation studies [21,22,23].

The cross-sectional image of the photothermal agarose microstructure was reported previously [24]. In this report, we showed the formation of a sharp rectangular agarose wall after irradiating a focused IR laser to the thin agarose layer. Hence the narrow and deep agarose microchannels can be formed within the resolution of the diffraction limit of optics.

## 4. Discussion

We evaluated the ability of agarose photothermal microfabrication technology and its microstructures for collective cell migration studies. We applied the photothermal technology for forming one to two mm sized cell reservoir chamber and straight microchannels from 10– 211 μm width in the thin agarose layer coated on the bottom surface of the cultivation dish. The results demonstrated that the agarose reservoir chamber and microchannels confined the cells and controlled the direction of collective cell migration within these confined structures effectively without any cells escaping, such as climbing over or entering under the boundary walls of agarose structures.

Adding to the ability to restrict cells in the structures, we also examined another advantage of the photothermal agarose microfabrication technology, which is the additional microfabrication during cell cultivation. As described in Figure 2c,d, microchannels were formed after the cells were filled in the reservoir chamber by proliferation to set the timing of collective cell migrations into microchannels near synchronously. The results also demonstrated that the additional microfabrication of microchannels using laser absorption did not induce apoptosis of cells in the reservoir. The cells in the reservoir started to proceed simultaneously into a series of different sizes of microchannels as soon as the microtunnels were formed at the edge of the reservoir chamber regardless of the width difference of channels. Thus, this evidence also supports that the agarose microfabrication is undestructive for collective cell migration assay. Hence, we concluded that the potential damage caused by the microfabrication of microchannels during cultivation was minimal in the experiments.

These results also suggest the potential and possibility of this flexible change of microstructures during cultivation as one of the next step technologies to assess collective cell migration inside spatially confined environments. For example, by exploiting the photothermal etching (during cultivation), the recovery of the wounded tissue model can be designed by abruptly changing the restriction area of the agarose reservoir chamber.

In this study, we also introduced some examples of measurement results during the examination of the ability of photothermal agarose microfabrication technology. Adding to the conventional findings of the collective cell migration, we confirmed the co-existence of two factors contributing to the propagation velocity of cells, the cell’s aspect ratio, which caused the velocity decrease in narrower microchannels, and the induced random walk-like behavior in wider microchannels. The mean propagation velocity has a peak around 30 μm width (as shown in Figure 4d), and decreased both in the narrower and the wider microchannels from this peak velocity. The mean migration velocity in microchannels smaller than 30 μm decreased as the aspect ratio of cell shapes increased, which is contrasting with the previous report explaining that the collective migration velocity becomes faster when the channel width becomes narrower under the condition of the width ranging from 20 μm to 400 μm of fibronectin patterns [9]. The difference may be a result of the confining material or cell morphology. In the typical fabrication technology attempting to control the width through fibronectin patterns, the aspect ratio of the cells appears less elongated than in our system, even in the narrowest width at 20 μm. Other reports also proclaimed a similar tendency of acceleration in propagation velocity for narrower microchannels [12]. However, here the cells in narrower agarose microchannels increased in aspect ratio, and the detailed single-cell tracking in our narrower agarose microchannels revealed the correlation between the increase of the shapes’ aspect ratio and deceleration of the cell. Agarose may have emphasized the increase of aspect ratio of cells in narrower microchannels especially for cases smaller than 30 μm, whereas other materials in previous reports may not have influenced the aspect ratio of cells in the range of widths of their experiments.

The tracking of single cells in wider microchannels also exhibited that the decrease of propagation velocity in collective cell migration was caused by the less coordinated movement of component cells. This is consistent with the previous reports [9,12] where the orientation of the cell was introduced as a crucial factor for the increase of the migration velocity in narrower microchannels. Hence, the decrease in velocity in narrower channels reported in our above results may not abide by the same principle of the previous reports and have not been observed before.

The presented results of the propagation velocity in narrower width microchannels are consistent with the hypothesized dominant mechanism of collective cell migration that the follower cells in the monolayer cells are regulated by the leading front cells [12]. In Figure 5, we demonstrated that the aspect ratio of single cells increased in narrower channels. Moreover, the front cells were the longest in this alignment and the follower cells were shorter (Figure 6).

As exhibited in the wider microchannels (Figure 8), we have observed a decrease in the end-to-end distance while the total displacement distance was maintained. The random walk-like behavior of cells in the two-dimensional cell sheets was already reported using PIV analysis of single-cells and concluded that the collective cell migration is a mixture of directional motion and diffusive motion [9,12]. This result is also similar to the emergence of cell swirls reported in the recent paper [25,26,27]. It includes the idea that the cells not only spread diffusively but also larger random walk-like fluctuation, in which the movement was occasionally against the direction of diffusion (see Figure 7), appeared in the wider microchannels.

The above results were the observation of collective cell migration in the open-ended confined geometries exploiting microfabrication technologies regardless of the contribution of extracellular matrix or hydrogels. As the stiffness of agarose gel is in the range of $1\;\times \;10^{-2}$ N/m^2^, the difference of stiffness of agarose microstructures against stiffer PDMS structures might have induced different behavior of collective cell migration [28]. However, to understand how tissues or organs attain their shapes, we need to consider the contribution of cell migration through the extracellular matrix (ECM) and its effect on the self-organization of cells into tubular structures [29,30]. Especially the importance of stiffness of ECM in cell migration and invasion was reported [31]. Tumor invasion in the thin confined geometry of ECM-based microchannels revealed that the migration velocity of cells in those thin microchannels was much faster than those in wider ECM microchannels caused by the increased polarization of cell-ECM traction forces, which increased as ECM stiffness rise [32]. When we take into account that the agarose walls are non-adhesive and thus do not allow for the attachment of stress fibers to walls, the results for the excessively thin agarose microchannels showing lower migration speeds might be explained.

In this report, we examined the advantages of photothermal agarose microfabrication technology for the application of collective cell migration study. As the next step of agarose microfabrication technology for studying collective behavior should be about the migration in more complicated structures and focusing on the interactions of cells when exposed to abrupt micropattern changes. These responses should elucidate further veiled rules that govern collective cell migration for more precise and practical model formation.

## 5. Conclusions

We applied the photothermal agarose microfabrication technology for collective cell migration measurement. The technology was successfully adopted and acquired the width-dependent propagation manner from 10 μm to 211 μm. The cells propagated from the reservoir remained inside the etched microchannels and no cells exceeded the etched structures even though a covering lid was not placed on the agarose microstructures. In the single-cell size microchannels, we observed that constriction in the environment can increase the aspect ratio of single cells. We also have demonstrated another advantage of agarose microfabrication, which is the flexible stepwise microfabrication even during cell cultivation, for regulation of initial starting timing of cell propagation after the cells were confluent in the reservoir. Exploiting the advantage of agarose microfabrication technology, agarose microchannels from 10 μm to 211 μm were fabricated with a spot heating of a focused infrared laser after the cell reservoir is filled by the cells. We measured the structural dependency of the front and follower cell velocities of epithelial cells. In straight channels, the velocity maintained constant regardless of their extension length, whereas the convex width dependency in velocity was observed; a peak velocity at around 30 μm in width and decreased both in wider and narrower channels. This width dependence can be explained by two rules; (1) velocity is decreased in the narrower width with the apparent increase of aspect ratio of cell shape, and (2) velocity is decreased by the increase of random walk-like behavior as width increases. Hence, we conclude that agarose microfabrication can be successfully applied to assessing collective cell migration and has shown great promise for more facilitated and flexible preparation of topologically confined environments for probing collective mechanical behavior.

## Figures and Tables

**Table 1:** Summary of photothermal agarose microfabrication technology.

Specification	Value
Spatial resolution (µm)	2
Fabrication speed (µm/s)	100
Modification during cultivation	◯
Damage on substrate surface	×
High-aspect ratio wall formation	◯

◯: Yes, ×: No.

## Supplementary Materials

The following are available online at https://www.mdpi.com/article/10.3390/mi12091015/s1, Video S1: MP4 time course 10 min interval 120 images of collective migration of epithelial cells from reservoir into the newly formed microchannels. Bar: 100 μm.
